# Building an initial programme theory to explain how and why on-the-day surgery cancellations occur and how they might be reduced

**DOI:** 10.1186/s12913-025-13592-x

**Published:** 2025-11-05

**Authors:** Buddhika S. W. Samarasinghe, Ross Millar, Mark Exworthy, Justin Aunger

**Affiliations:** 1 Health Services Management Centre, School of Social Policy and Society, Park House, University of Birmingham, Birmingham, B15 2RT UK; 2 NIHR Midlands Patient Safety Research Collaboration, University of Birmingham, Birmingham, UK

**Keywords:** Surgery cancellations, Realist evaluation, Realist theory building, Multi-methods

## Abstract

**Background:**

On-the-day surgery cancellations (OTDSCs) have been a longstanding global problem, bringing significant suffering to patients and carers, and substantial waste across healthcare systems. Any cancellation of a surgery that occurs for any reason on the day of the scheduled surgery is defined as an OTDSC. Despite the high prevalence of OTDSCs, little is known about why they happen and how to minimise them. This study aimed to develop Initial Program Theories (IPTs) and share valuable insights that can form the basis for future evaluation of OTDSCs.

**Method:**

We conducted a study to address the questions, “How do OTDSCs occur, and in what contexts can they be minimised?“. We used a qualitative and multi-stage approach to developing IPTs. Data collection included OTDSC literature (*n* = 35) identified from a systematic search, including feedback sessions with administrators (*n* = 10) from eight NHS trusts, two feedback events with patient expert advisers (*n* = 6), and expert practitioners (*n* = 8).

**Results:**

The iterative analysis found that OTDSCs are a complex undesired outcome, influenced by many interconnected “variables” at macro-level (e.g., waiting-list policies, austerity measures and workforce shortages) and meso-level (e.g., workload, high emergency admissions and interruptions), as well as healthcare professionals’ (HCPs) and patients’ perceptions and behaviours. The study identified that failures in various aspects of individualised care (such as care planning, communication and resource allocation) in preparing for surgery before admission could also contribute to different types of OTDSCs.

**Conclusion:**

As a result of the complex and interconnected nature of OTDSCs and the wide variety of causes, it can be hard to reduce their occurrence. OTDSCs can be minimised by carefully considering various aspects of individualisation of care, such as clinical care planning, communication and resource allocation and delivery when preparing patients to undergo surgery. Providing favourable working conditions and creating effective knowledge transfer between the stakeholders initiating OTDSCs and HCPs who prepare patients for surgery can be critical to minimising most OTDSCs. The study developed a taxonomy and novel IPTs that have practical implications for policymakers and practitioners when designing interventions to minimise OTDSCs.

## Background

Evidence suggests that On-the-day surgery cancellations (OTDSCs) are a significant and longstanding problem faced by healthcare providers, policymakers, patients and HCPs. Most surgeries are completed using scheduled surgical care systems (SSCSs) that manage surgical care from patient preparation into surgery, and to recovery [1]. OTDSCs are an unintended outcome of SSCSs, which include various conditions, including cancer, and the prevalence of these is quite high. OTDSCs are an unintended outcome of scheduled SSCSs, which include various conditions, including cancer, and the prevalence of these is quite high. For example, the global prevalence of case cancellation on the intended day of surgery has been found to be 18%, according to a meta-analysis [1]. The economic and psychological burden of OTDSCs for patients and families is well documented and emotionally harmful to patients, with a series of negative effects, including anxiety, anger, rejection and physical/psychosomatic symptoms in the extended waiting period [2].

To improve the efficiency and experience of scheduled care waiting lists, policymakers have increasingly relied on standardised interventions such as performance targets, care pathways, protocols, standard operating procedures and checklists to manage patients and resources. Yet in recent years, the UK National Health Service (NHS) has exemplified how policy decisions such as austerity measures, staffing shortages, increasing emergency admissions and inadequate social care resources, have created problems for hospitals in delivering effective and safe surgical care. Furthermore, NHS hospitals are government-funded systems where patients with varying clinical complications, multiple co-morbidities and diverse socio-economic needs (i.e., among people of different income groups and ethnicities) undergo scheduled care surgery. There are also inequalities in those who experience OTDSCs. Patients undergoing surgery with few clinical complications are less likely to experience OTDSCs for clinical reasons [3–5]. Evidence from the UK [6], the US [7] and Singapore [8] suggests that OTDSCs are common among patients from disadvantaged communities (e.g., low-income groups and homeless people). Similarly, a wide range of evidence highlights that patients who undergo surgery with high levels of clinical complications are more likely to report OTDSCs [7, 9].

As a result, OTDSCs have been a significant problem in the English NHS [6, 10, 11]. A large cohort study found that 13.9% of patients attending inpatient operations were cancelled on the day of surgery. The biggest reason for OTDSCs is that patients are unfit for surgery, accounting for 33.3% of OTDSCs [12] Reported loss of income from OTDSCs in hospitals can be substantial, and, in the English NHS, the cost of lost operating theatre (OT) time because of surgery cancellation is as high as £400 million per year [13].

Delivering safe and efficient surgery has become a significant global health challenge [14]. Surgery is a high-risk invasive procedure completed under local or general anaesthetic (GA), mostly performed in an OT, which can have significant benefits for patients, including saving lives, relieving pain and improving quality of life [15]. There are two main types of surgeries: emergency and scheduled care. In most healthcare systems, millions of patients have been waiting for surgery for more than a year, and SSCSs are under extensive pressure to meet growing demand and deliver high-quality, low-cost care [16, 17]. Care delivery in SSCSs varies significantly because of various contextual factors, including care settings (e.g., university, district, general and veterans’ hospitals), the nature of the payer organisation (e.g., insurance company, government), and legal and regulatory frameworks [7, 11, 13]. To capitalise on the scheduled nature of care, most SSCSs use a series of interventions to prepare patients clinically and emotionally for surgery and allocate resources for surgery before admission. Every patient undergoing surgery has unique clinical and social needs; SSCSs use different types of standardised interventions to prepare patients for surgery. For example, standardised clinical care planning interventions (e.g., pre-operative care assessments, protocols, and pre-assessment tools) are used to identify and mitigate various clinical risks (e.g., existing health conditions, allergies) and prepare patients for surgery.

OTDSCs happen for various reasons, often shaped by local contexts, making them harder to understand and minimise. Studies have used a wide range of subjective and atheoretical categorisation of OTDSCs, incorporating categories such as DNA (Did Not Attend), patients deciding not to provide consent, patients being unfit for surgery, and the unavailability of physical (e.g., post-operative beds, theatre equipment and medication) and personnel resources (e.g., surgeons, anaesthetists and theatre practitioners) [7, 11, 18]. The most common reason for OTDSCs is, arguably, patients being unfit for surgery [7, 11, 18]. This can be because of failure to manage one or more of a patient’s clinical risks related to co-morbidities – such as anaemia, diabetes, obesity and kidney disease – or because of historical infections, cardiac problems or medication [11, 18]. For example, this category includes reasons such as patient medication not being correctly managed or stopped (e.g., anti-coagulant medication), patient co-morbidities not being well-managed (e.g., anaemia, hypertension), patients’ relevant tests not completed or available (e.g., blood tests, X-ray), and patient allergies (e.g., latex, antibiotic) not identified [4, 8, 19]. Other highly reported reasons include patients being marked as DNA and patients failing to provide consent [9, 20, 21].

Many OTDSC categorisations are subjective, and the number of reasons varies significantly [18, 20, 22, 23]. On some occasions, reported reasons for OTDSCs varied significantly within organisations. Such variation and confusion among organisations and studies about how to categorise OTDSCs is a barrier to developing evidence to minimise OTDSCs and compare OTDSC rates within and outside a given organisation. Most importantly, the inability to categorise OTDSCs misses the opportunity to identify areas of improvement in the surgical care pathway. To overcome the limitations and consider the complexity around OTDSCs, developing a typology of OTDSCs and a reporting tool informed by realist analysis would aid practitioners to understand OTDSCs better, why they occur, and how they might be minimised.

### Research questions

Although the significant negative impact on patients and organisations (in the form of, for example, waste of resources) is widely acknowledged, the literature on OTDSCs largely consists of atheoretical descriptive studies from a wide range of care systems without published systematic, realist, or economic impact reviews [7, 11, 18]. Studies to date have used various atheoretical and narrowly defined approaches to categorise reasons for OTDSCs [4, 7, 24], which is a significant barrier to developing interventions to minimise them [7, 18, 20]. As such, little is known about the interrelationships surrounding why OTDSCs occur or how to minimise them.

This study is the first phase of a realist review to answer the research questions:

1. How can OTDSCs be categorised in a wide range of SSCSs?
2. How and why are OTDSCs happening, and in what contexts and to what extent can they be minimised?
3. How can reporting of OTDSCs be improved?

The scope of the paper is limited to the understanding various insights that help to develop IPTs. In the next phases of this research outside this paper’s scope, the developed IPTs will be further refined using empirical and literature data.

## Methodology

### The rationale for the use of realist methods

A realist approach is suitable for understanding OTDSCs due to their complexity. Realist reviews are well suited to studying complex phenomena such as OTDSCs, where it is recognised that various social and economic contexts influence phenomena and interventions [25]. A realist review is a theory-driven approach that goes beyond whether an intervention works to consider how and why it works, for whom, and in what circumstances [16, 17]. The aim of a realist review is to develop - and later to refine, refute and confirm - programme theories to explain the aforementioned workings of intervention(s). OTDSCs are embedded in interconnected structures that epitomise complexity, where multiple layers of contexts contribute to their occurrence. Understanding and producing evidence that could help prevent such outcomes, in the form of programme theories, may help us to understand how context can be tweaked to prevent OTDSCs in the future. Pawson argues that realist methods provide a coherent methodological approach to tackling complexity while appreciating context sensitivity and producing transferable knowledge using theory-based evaluation [25, 26]. The realist approach can use both qualitative and quantitative data [26]. Another advantage of using the methodology is the ability to use diverse international evidence from various care environments from a wide range of disciplines and methods (i.e., qualitative and quantitative) related to OTDSCs.

Realist reviews have historically used numerous methods to develop IPTs, providing the initial foundations of realist investigation [27]. Examples include using ‘known’ literature to study teams and stakeholder workshops [28]. Without pre-existing well-developed and theoretical literature-related OTDCSs, the study design aimed to synthesise data from various sources using multiple methods approaches to develop IPTs as part of a realist review. Patients, clinicians and hospital administrators are closely involved in OTDSCs. Therefore, in addition to the literature, we decided to gain their insights using various feedback methods from patients, clinicians and hospital administrators to develop theories. This study adhered to the RAMESES guidance and publication standards for realist syntheses [29].

### Study design and data collection

The IPT building process was completed across three phases. In phase one, preliminary IPTs were identified from OTDSC literature (*n* = 35). The studies were identified using a systematic literature search (see Table 1) and appraised using a mixed-method appraisal tool (MMAT). The preliminary IPTs identified in phase one were further refined using multiple methods, which involved gaining feedback from various experts, including administrators, practitioners, and patients. All the data collection, including the literature reviewed, were completed between January 2019 and February 2020 (i.e., before hospitals were affected by the COVID-19 pandemic). During this time, NHS hospitals in England were severely affected by austerity measures, high emergency admissions and delayed discharges.

### Literature searches

The literature review in phase one involved three types of searches to identify relevant material: conventional database searches, searches of selected websites, and supplementary searches The following databases were used to search for studies: Scopus, Cochrane and Cinahl Plus. Searches were limited to human studies and papers published in English without limiting them to a time period. Further website searches were carried out using keywords relating to the Social Care Institute for Excellence, The King’s Fund, NHS Improvement and the Nuffield Trust. These website searches used the search functions available on the websites concerned.

Several search strategies were developed and revised several times using advice from information specialists. The search strings focused on various terms related to surgery (e.g. elective procedure or activity), cancellation (e.g., terminate or rescheduled) and place of care (e.g., hospital or acute care). The final version of the search strategy is described in the table below (see Table 1).

**Table 1:** Summary of search strategies used for databases

Search query used	“surg*” OR “elective activit*” OR “elective procedure*” OR “operation*” AND “cancel*” OR “terminate*” OR “reschedule*” OR “abandon*” AND “health*” OR “hospital*” OR “acute care”
Database searched	Web of Science, Scopus, Cochrane, Cinahl Plus, Medline
Exclusion criteria	Non-human studies Languages other than English

All database searches were conducted during July 2019 and updated in November 2019. The inclusion criterion was “Could this article provide useful information about transferable patterns or trends that understand or minimise OTDSCs?”. All study designs were included that were useful for theory generation.

During the full paper screening, purposeful sampling was used to capture studies from various care settings and research designs to develop exploratory and transferable IPTs. The rationale for using a purposeful sample was to embrace the richness and breadth of available literature on OTDSCs. For all the empirical studies (except the evidence review), the quality was appraised using the mixed method appraisal tool (MMAT) [29] and this was used to inform judgements regarding rigour about included literature from a realist perspective in line with guidance [30].

### Primary data collection

The use of multiple methods that focused on several different actors (such as administrators, HCPs, managers from different disciplines and patients) helped to capture different perspectives to answer the research questions. This included feedback sessions with administrators (*n* = 10) from eight NHS hospital trusts as part of the feasibility study, two feedback events with patient expert advisers (*n* = 6), and eight expert practitioners (*n* = 8). Table 2 illustrates the overview of three methods.

**Table 2:** Overview of method and findings used to develop IPTs

Method	Overview of method
Feedback from administrators as part of the feasibility study	The main data collection aim was to understand how NHS administrators and leaders viewed and managed OTDSCs. The feasibility study also provided an important platform to communicate and engage with a wide range of NHS administrators (*n* = 10) from eight trusts. The feedback focused on why different types of OTDSCs are happening and how they report them in their information systems. Ten senior leaders/administrators with significant experience in tackling and minimising OTDSCs include three Head of Operations in theatres, four General Managers, and three Service Managers.
Two feedback events with patient representatives (*n* = 6)	Patient representatives were chosen as they tend to have a broader understanding of the challenges patients face undergoing surgery. The data collection focused on understanding patients’ perspectives on minimising OTDSCs using patient experts’ views. All six patient experts worked in different NHS organisations as volunteers and had many interactions and experiences with various patient cohorts, including patients who have experienced OTDSCs. Two feedback events focus on why OTDSCs are happening and how to minimise them. Each session lasted around one and a half hours, excluding the break. The information was captured in flip charts, and notes were taken.
Theorising with eight expert practitioners	The feedback exercise involved the researcher using a series of why questions to understand how various concepts and interactions contributed to different types of OTDSCs. The preliminary IPTs were presented to expert practitioners. This approach helped to understand the inner workings of various standardised interventions. All the theorising exercises were completed as a fact-finding mission. All the information is captured as a diagram using notes. Practitioners with more than ten years of experience in surgery were involved in the exercise. Five clinical (one surgeon, two PONs and an anaesthetist) and three non-clinical (middle-level managers) expert practitioners were involved in the exercise.

### Ethical considerations and registration

The wider realist project received ethical approval from the University of Manchester and the Health Research Authority in the UK. The realist review protocol is registered with the International Prospective Register for Systematic Reviews (PROSPERO) database [Ref: CRD42019124272].

### ICAMO heuristic tool

The traditional CMO (context–mechanism–outcome) heuristic tool developed by Pawson and Tilley was expanded to include “intervention” (I) and “actors” (A) to develop the ICAMO heuristic tool [26]. This was used to formulate more precise IPTs in this study. The use of ICAMO heuristic tool helps to gain a detailed understanding how various actors (PONs, surgeons) contributed to interventions within various contexts. This technique was chosen over regular CMOCs due to our particular findings during taxonomy development which highlighted the importance of OTDSC initiation by relevant actors. The ICAMO heuristic tool was used to organise and analyse data to identify connections between each component in the tool and provide explanations. The elements of the ICAMO are illustrated in Table 3 with examples related to SSCSs. The definitions of the elements were adopted from another realist review [26].

**Table 3:** Definitions of the elements included in the ICAMO heuristic tool

Elements included in the ICAMO heuristic tool	Definition	Examples from SSCSs
Interventions	A combination of programme components designed to produce behaviour changes or improve health status among individuals or group members.	Standardised interventions include pre-assessment tools, clinical protocols, patient interview guides and scheduling tools.
Context	The salient conditions that are likely to enable or limit the activation of mechanisms.	Various NHS policies (e.g., waiting list targets and austerity measures) and organisational conditions (e.g., high workload and inter-professional collaboration).
Actors	The individuals, groups and institutions that play a role in implementation and outcomes.	Pre-operative nurses (PONs), doctors, anaesthetists and surgeons.
Mechanism	Any underlying determinants of social behaviour generated in certain contexts that are hidden and not easily identifiable or measurable but real.	Patients’ trust in the clinical team, compassion among anaesthetists towards patients undergoing surgery.
Outcomes	Expected (i.e., desired outcomes) or unexpected effects (i.e., undesired or desired outcomes, additional outcomes) of interventions.	Individualisation of care related to clinical preparation of patients undergoing surgery (desired outcomes) and individualisation of care related to resource preparation related to undergoing surgery (desired outcomes), OTDSCs, delayed discharges (undesired outcomes).

### The journey to develop IPTs

We developed IPTs through an iterative process consisting of a multi-stepped approach to gleaning, refining, and refuting theories. The initial phase drew upon the literature outlined earlier. For each study, data were extracted into a table consisting of eight columns: reference, title of the study, country, information related to care setting, study design, main findings, and how the intervention may influence OTDSCs (i.e., applying “realist logic”). Data from these studies helped to form initial ideas around the context within which OTDSCs are being understood and what types of OTDSCs there are. This helped to develop an initial version of the typology of OTDSCs outlined later.

The second phase involved understanding how NHS administrators and leaders viewed and managed OTDSCs, when we engaged with a wide range of NHS administrators (*n* = 10) from eight trusts. Focuses of these discussions were on why different types of OTDSCs are happening and how they report them in their information systems. This helped understand the wider macro-context within which Trusts are operating.

Our third phase involved speaking to patients to understand patient-side contributors to OTDSCs with six patient contributors. The data collection focused on understanding patients’ perspectives on minimising OTDSCs using patient experts’ views.

The final step involved direct theory refinement and refutation with eight expert healthcare practitioners using presentation of rough theories, and a series of ‘why questions’ to understand how various concepts and interactions contributed to different types of OTDSCs. This approach helped to understand the inner workings of various standardised interventions and produce IPTs presented later here. We then revisited all the data gathered across all sources to test the IPTs and perform a final ‘sense check’.

### Findings

#### Results of study selection

In total, 3,316 articles were retrieved from databases and websites. We excluded 3105 articles for broad reasons, including a lack of relevance to the topic and dublications, leaving 211 articles for full-paper screening. Most of the excluded articles explain cancellations or rescheduling procedures rather than the surgery itself, such as procedures completed in out-patient clinics, radiotherapy treatments, and various diagnostic tests (e.g., X-ray, CT scans). Based on the inclusion criteria 35 articles were identified. Among the 35 studies, the designs varied, including 15 quantitative descriptive [6, 9, 11, 12, 18–20, 22, 23, 31–36] (e.g., incident prevalence studies and survey case reports), nine quantitative non-randomised control [4, 5, 8, 37–42] (e.g., cohort studies, case-control studies and cross-sectional studies), five evidence reviews [10, 21, 43–45] (e.g., literature reviews, commentaries and editorial pieces), one randomised control study [46], three mixed-method studies [7, 47, 48] and two qualitative study [49, 50]. The 35 studies came from 12 countries, emphasising that OTDSCs are a global problem. Most studies were from the UK (*n* = 7) and the United States (US) (*n* = 7).

### Taxonomy of OTDSCs and IPTs

The inability of standardised interventions to provide individualised care for each patient undergoing surgery is an important context influencing the creation of OTDSCs. Failure to prepare patients and address their individualised care needs before surgery using these interventions provides the context to increase the likeliness of OTDSCs. The analysis identified three main actors responsible for initiating most OTDSCs: clinicians (usually anaesthetists or surgeons) who assess clinical suitability for the surgery, patients undergoing surgery, and administrators responsible for resources. For example, an inadequate level of individualisation of care in clinical care planning can lead the clinicians responsible for surgery to decide to cancel the surgery for clinical reasons. Similarly, an inadequate level of individualisation of care relating to resources can lead to OTDSCs being initiated because of administrators’ lack of resources (e.g., post-operative beds and professionals). In contrast, when patients undergoing surgery do not meet their individualised communication needs, patients initiate OTDSCs themselves (for reasons such as DNA and withdrawal of consent for surgery).

Patients undergoing surgery have diverse set-up needs (i.e., clinical, social and economic resources) and staff may improperly identify and address important aspects of individual patient needs in preparing patients for surgery. This leads to different types of OTDSCs [3, 40, 42]. In real care settings, standardisation efforts can reduce the individualisation of care, too. For example, standardisation interventions such as clinical care planning, communication and resource allocation (i.e., scheduling), can create various interconnected events and lead to different types of OTDSCs [10, 24]. Different aspects of individualisation of care – clinical care planning, communication, and resources, related to different types of OTDSCs. Table 4 provides the details of four IPTs that cover different types of OTDSCs. IPT one, two, and three are linked to standardised interventions to prepare for surgery by PONs while recognising the interplay between standardisation and individualisation of care and its influence on OTDSCs.

The analysis found that various aspects of inadequate of individualisation of care provide to different types of OTDSCs which is one of the main focused of the IPTs. The taxonomy below explained how different types of individualisations of care leads to different OTDSCs initiated by actors. For an example, inadequate level of individualisation of care inpatient communications leads to patient initiated OTDSCs.

**Fig. 1:**
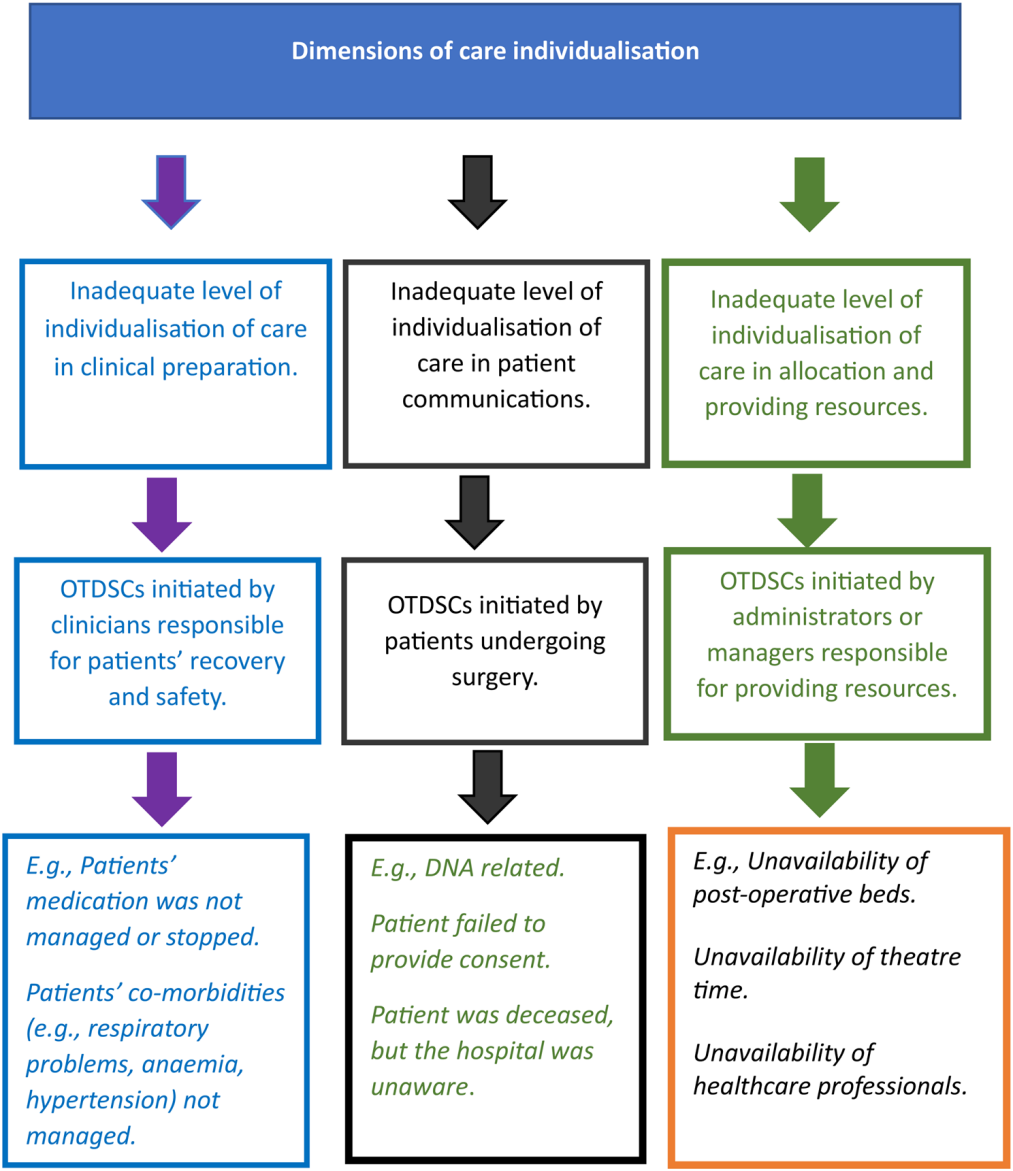
Taxonomy related to various reasons for OTDSCs and why different actors initiate OTDSCs

#### Initial programme theories

In practice, various standardised interventions (care planning, communication and delivery) used in delivering care leads to different type of OTDSCs which provide the basis for the IPTs. Following provides the building blocks to the IPTs: behaviours of various actors, organisational contexts, policy implications, various types of standardisations and individualisation of care. Table 4 depicts all the IPTs.

**Table 4:** Details of four IPTs developed from multiple evidence sources

Title of IPT	IPT overview
One –OTDSCs because patients are deemed unfit for surgery.	When care is delivered using standardised clinical care planning interventions (i.e., checklists, clinical protocols, pre-assessment tools) to prepare patients for surgery, the ability of PONs (actors) to make confident decisions (mechanism) is negatively influenced by various unfavourable working conditions (meso-contexts). As a result, inadequate levels of individualisation of care in clinical preparation (outcome/context) leads to OTDSCs because patients are perceived as unfit for surgery (main outcome), initiated by clinicians who review patients on the day of surgery. Unfavourable organisational conditions include lack of time to complete clinical consultation, high levels of interruptions to clinical work because of emergencies, high workload and inadequate staffing.
Two –OTDSCs for reasons such as patient DNA or refusal to undergo surgery.	When care is delivered using standardised communication interventions (i.e., pre-interview protocols, text, communication checklists and follow-up calls), various unfavourable working conditions (meso-contexts) negatively influence the ability of PONs (actors) to communicate with patients. As a result, this leads to an inadequate level of individualisation of care in patient communication (outcome), resulting in lack of development of patient trust (mechanism) in the clinical team and leading to OTDSCs being initiated by patients for reasons such as DNA and withdrawing consent (main outcome). Unfavourable organisational conditions involve a lack of time to complete clinical consultation, high levels of interruptions to clinical work for emergencies, high workload and inadequate staffing (meso-contexts).
Three –OTDSCs resulting from resource unavailability: scheduling.	When care is delivered using standardised scheduling interventions (i.e., scheduling tools based on historic theatre time), various unfavourable working conditions negatively influence the ability of PONs (actors) to make confident decisions (mechanism). As a result, inadequate levels of individualisation of care relating to allocation based on clinical needs lead to OTDSCs by administrators responsible for providing resources (various types of post-operative beds or staffing) (main outcome) Unfavourable organisational conditions include resource unavailability, lack of inter- and intra-professional collaboration, high workload and inadequate staffing.
Four –OTDSCs resulting from resource unavailability: care delivery.	When care is delivered using standardised protection interventions (i.e., dedicated theatres for emergency and ring-fenced beds), the ability of HCPs and administrators (actors) to deliver resources by addressing individual patients’ clinical needs (outcomes) is negatively influenced by various unfavourable working conditions. As a result, an inadequate level of individualisation of care when providing resources based on patients’ clinical needs can lead to OTDSCs initiated by administrators responsible for providing resources (managers responsible for various types of post-operative beds, staffing or equipment) because of resource unavailability (main outcome). Unfavourable organisational conditions (meso-contexts) involve a lack of inter- and intra-professional collaboration, high workload and inadequate staffing.

#### IPTs relating to pre-operative care (category one)

Category one: Failure to identify various clinical, communication and resource allocation needs linked to preparing patients for surgery (i.e., pre-operative care). This category includes OTDSCs that occur due to failure to prepare patients for surgery due to clinical care planning, communication or resource allocation. For example, failure to prepare patients by managing various clinical factors (e.g., anaemia, allergies, hypertension, medication or lack of diagnostic results) as part of clinical care preparation can lead to patient OTDSCs for clinical reasons [7, 41, 47, 50]. This encompasses IPT 1, 2 and 3, outlined below.

Initial programme theory 1: OTDSCs because patients are deemed unfit for surgery.

The first IPT explains how standardised clinical care planning interventions failed to individualise care due to various organisational and national contexts that can lead to OTDSCs due to clinical reasons (i.e., patients are unfit for surgery). For example, when patient individualisation is related to clinical planning (e.g., lack of blood tests, failure to stop anti-coagulation medication and managing co-morbidities), clinicians (usually anaesthetists) responsible for surgery can decide to cancel them on the day of the surgery for clinical reasons. This could include avoiding any potential harm or patient safety issues or to protect themselves or their institution from litigation.

Initial programme theory 2: OTDSCs for reasons such as patient DNA or refusal to undergo surgery.

The second IPT explains how standardised communication interventions fail to individualise care due to various organisational and national contexts that can lead to OTDSCs due to patient reasons. Patients undergoing surgery usually feel anxious and have diverse communication needs; greater individualisation can help mitigate this. Some of their anxiety is related to previous experiences with HCPs [50]. Unmet communication needs in preparing patients for surgery before they are admitted leads to OTDSCs for reasons such as DNA, withdrawal of consent, or patients requesting time to think about side effects. All these reasons for OTDSCs could be minimised, and patients could be better prepared for surgery if their communication needs were understood and met.

Initial programme theory 3: OTDSCs resulting from resource unavailability in scheduling.

The third IPT explains how standardised resource allocation interventions failed to individualise care due to various organisational and national contexts leading to OTDSCs due to resource unavailability. Based on patients’ individual clinical and socio-economic reasons, they require various resources, such as medication, blood, equipment and post-operative beds, specialised professionals and patient discharge requirements. When hospitals do not have specific resources based on individual patient needs to complete surgery, they are likely to experience OTDSCs because of resource unavailability [4, 21, 39, 51]. After patients are admitted for surgery, administrators (bed and theatre managers) assess their ability to carry out surgery safely based on resource availability and ability to provide intra- and post-operative care. Unavailability of resources for surgery can happen for category one and two reasons: inadequate levels of individualisation of care that are related to scheduling resources (e.g., failure to identify specific post-operative beds or specialist professionals) and unavailability of resources on the day of the surgery (e.g., inpatient post-operative beds given to emergency patients).

#### IPTs relating to failures in care delivery (category two)

This category includes OTDSCs occur due to various failures related to care delivery. For example, although in pre-operative care resources are allocated based on patient needs in advance, they cannot be delivered because of their unavailability on the day of surgery – resources sometimes end up being reallocated to other patients because of resource prioritisation. For example, post-operative beds supposed to be allocated to scheduled surgical care patients might be prioritised and given to emergency patients. This is described as unavailability of resources (e.g., theatre time or post-operative beds). Across all types of collected data (i.e., literature and feedback), OTDSCs resulted from post-operative bed unavailability driven by failure to deliver care (i.e., category two) primarily because of a lack of social care, delayed discharges and high levels of emergency admissions [4, 12]. These dynamics are reflected in IPT 4.

Initial programme theory 4: OTDSCs resulting from resource unavailability in care delivery.

The fourth IPT focuses on interventions protecting resources allocated to scheduled surgical care patients from being diverted to emergency care patients, such as dedicated emergency theatres and ring-fenced beds. The main actors related to the fourth IPT are various HCPs and administrators involved in managing the patient flow of scheduled care and emergency care, such as matrons, clinical leaders, flow managers, and bed managers.

### Macro-context: external shocks and resource allocation

OTDSCs across both categories are common. Often, NHS hospitals’ failures to deliver care are outside the hospitals’ control [4, 12], because of, for example, infection outbreaks in neighbouring hospitals, high levels of emergency admissions because of a flu outbreak, or critical accidents leading to increased emergency admission levels (e.g., multiple accidents or bomb blasts) [4, 40, 52]. In contrast, adequately preparing patients for surgery and improving individual clinical, communication and resource allocation before admission (category one) largely depends on the hospital’s ability to do this. These two categories are linked to different parts of the care pathway, and interventions to minimise them also vary. OTDSCs related to category one can be minimised by improving pre-operative care interventions. In contrast, category two OTDSCs can be minimised by improving patient flow and creating protection for scheduled surgical care to be carried out, in the form of, for example, dedicated theatres or post-operative beds (i.e., dedicated day cases or intensive care wards).

### A key demi-regularity: individualisation versus standardisation of care

We identified a tension between individualisation and standardisation of care across both the empirical and literature aspects of our study. Patients undergoing surgery have diverse set-up needs (i.e., clinical, social and economic resources) and staff may improperly identify and address important aspects of individual patient needs in preparing patients for surgery. This leads to different types of OTDSCs [3, 40, 42]. In practice, sometimes standardisation efforts can reduce the individualisation of care, too. For example, standardisation interventions such as clinical care planning, communication and resource allocation (i.e., scheduling), can create various interconnected events and lead to different types of OTDSCs [10, 24]. Different aspects of individualisation of care – clinical care planning, communication, and resources, related to different types of OTDSCs. Table 3 provides the details of four IPTs that cover different types of OTDSCs. IPT one, two, and three are linked to standardised interventions to prepare for surgery by PONs while recognising the interplay between standardisation and individualisation of care and its influence on OTDSCs.

We found that utilising evidence from multiple methods helps to understand the OTDSCs from different perspectives (Table 4). The practitioner experts agreed with administrators’ and patients’ feedback on how the tension between standardisation and individualisation of care and various organisational contexts (e.g., short time slots for clinical consultation, emergency interruptions, and high workload among PONs) leads to OTDSCs (IPT 3).

*The problem is that we have a single standardised care pathway where every surgical; patient goes through*,* and the same set of tools and documentations completed using the same protocol [standardised intervention]. Every patient is different plus unique*,* and this standardisation do not work*,* sometimes. Some complex liver cases require more comprehensive and individualised (outcome) work up in pre-op [pre-operative assessment]; otherwise*,* we get many complications like on the day of the operation and theatre delays. (Surgeon*,* p. 8)*

The experts also confirmed that various organisational contexts negatively influenced PONs’ clinical decision-making ability, leading to inadequate individualisation of care and resulting in OTDSCs. For example, failure to identify or mitigate clinical risks when using clinical care planning interventions leads to inadequate individualisation of care in clinical preparation, resulting in OTDSCs for clinical reasons (IPT 1). Some expert practitioners highlighted that surgical care is highly standardised (i.e., factory-like production line), which creates “tick box” cultures rather than addressing individualisation care needs. Consequently, this leads to various types of OTDSCs.

*The problem is we have too much pressure from CQC*,* NHS England plus the management (meso-context) to achieve cost reductions and waiting-list targets…Especially*,* pre-op nurses[PONs] have been under lots of pressure… they are badly affected by very high workloads and not tick to care*,* instead they do is ticking-boxes. This is the real cause of all these surgery cancellations [OTDSC] (Anaesthetist*,* p. 8)*.

On some occasions, we found that various organisational contexts influenced by policy directives increased the tension between standardisation and individualisation of care [53]. For example, the expert practitioners’ comments suggest that in response to national policy (e.g., austerity measures and achieving national waiting list targets), NHS hospital managers have had to increase PON workloads and limit pre-operative assessment to 15–20 min to improve waiting list times. This is reflected across the macro-context of the IPTs.

*The whole hospital is affected by emergency pressures*,* austerity measures and cost improvement initiatives…If you look at it over the years*,* to the pre-op [pre-operative] nurse’s job has become a form-filling admin role to save money … they have complete endless protocols*,* checklists and forms (standardised interventions) instead of [a] caring profession providing individualisation care… They [pre-operative nurses] have extremely busy clinics with 10–15 min clinic slots…So much standardised documentation and no time for understanding the patient’s needs (outcome)*,* so leading on the day surgery cancellations [OTDSCs]. (Theatre Matron*,* p. 7)*

PONs from several organisations had their clinical work interrupted because of high levels of emergency admissions and patient flow problems in hospitals. As a result, PONs have felt exhausted and stressed managing their workloads.

*Accident and emergency care problems (e.g.*,* high admission and overcrowding) create lots of interruption to pre-op [pre-operative] nurses*,* plus create high workload. They cannot do their nursing job the way they want because of interruptions…they get exhausted and stressed with emergency problems… it is too much pressure for them [pre-operative nurses] to their real job*,* this is why we have so many on the day cancellations [OTDSC] (main outcome) due to pre-op [pre-operative care] problems. (Theatre Practitioner*,* p. 14)*

All these factors have contributed to failures in preparing patients and addressing their individualised needs, consequently leading to various types of OTDSCs, because, for example, patients are unfit for surgery or have failed to provide consent (IPT 1/2), or there has been a failure to schedule resources (e.g., equipment, post-operative beds and specialist professionals) based on patients’ clinical needs (IPT 3).

## Discussion

Going through scheduled surgery is an anxious and vulnerable experience, and the risk of OTDSCs adds a substantial amount of anxiety to patients and carers. Despite significant work pressures and resource limitations (i.e., shortages of staff, discharge problems and lack of post-operative beds), most HCPs and leaders in the NHS appear to be trying their best to minimise OTDSCs in their local contexts. The findings of the heterogeneous literature review suggested that many OTDSCs are preventable [7, 11, 18].

Despite their complexity, OTDSCs are largely preventable. Most reported OTDSCs relate to patients being inadequately prepared for surgery (i.e., failures in pre-operative care). Most OTDSCs initiated by clinicians and administrators can be understood as a necessary response to minimise patient harm. For example, when clinicians who review patients undergoing surgery believe that a patient is not adequately prepared for surgery, they (mostly anaesthetists) tend to initiate OTDSCs for clinical reasons (i.e., patient unfit for surgery) because they believe undergoing surgery may lead to various patient implications (anaesthetic complications or harm, or delayed recovery).

Despite the complexity of OTDSCs, we found that most can be divided into two categories based on their links to different parts of the scheduled surgical care pathway.

Category one: Failure to identify various clinical, communication and resource allocation needs linked to preparing patients for surgery (i.e., pre-operative care) and Category two: OTDSCs occurring due to various failures related to care delivery Both categories are common. Often, NHS hospitals’ failures to deliver care are outside the hospitals’ control [4, 12], because of, for example, infection outbreaks in neighbouring hospitals, high levels of emergency admissions because of a flu outbreak, or critical accidents leading to increased emergency admission levels (e.g., multiple accidents or bomb blasts) [4, 40, 52]. In contrast, adequately preparing patients for surgery and improving individual clinical, communication and resource allocation before admission (category one) largely depends on the hospital’s ability to do this. These two categories are linked to different parts of the care pathway, and interventions to minimise them also vary. OTDSCs related to category one can be minimised by improving pre-operative care interventions. In contrast, category two OTDSCs can be minimised by improving patient flow and creating protection for scheduled surgical care to be carried out, in the form of, for example, dedicated theatres or post-operative beds (i.e., dedicated day cases or intensive care wards).

This paper developed four IPTs that explained several different types of OTDSCs connected to these two categories that might be used to design various interventions minimise OTDSCs. These IPTs explain how and why various standardised interventions related to clinical care communication, and resources lead to different types of OTDSCs due to [1] patients being unfit for surgery [2], patient DNA or refusal [3], scheduling issues and [4] resource constraints in care delivery. Initiated by different actors, such as clinicians, patients, and managers [18, 24, 40, 50]. To overcome the barrier of atheoretical OTDSCs, we also developed a taxonomy of OTDSCs (Fig. 1). We found that various aspects of care individualisation can be initiated by three types of actors: clinicians, patients, and administrators (Fig. 1).

A key tension or demi-regularity identified across IPTs was between individualisation and standardisation of care; either could lead to OTDSCs in different contexts. The literature suggested that SSSCs use various standardised interventions to minimise different types of OTDSCs. For example, PONs clinically prepared patients for surgery using standardised clinical care planning interventions (e.g., clinical care planning protocols, checklists for care planning and screening tools) [3, 42]. Similarly, resources (e.g., theatre time, equipment and professionals) were allocated to patients using standardised scheduling interventions (e.g., standardised scheduling tools, historical use of theatre times) [24]. The literature suggested that failure of these standardised interventions leads to different types of OTDSCs. For example, failure of standardised clinical care planning interventions to prepare patients leads to OTDSCs for clinical reasons (IPT 1). In contrast, strengthening standardised clinical care planning interventions can minimise OTDSCs for clinical reasons [7, 41, 47, 50].

### Implications for practitioners and policymakers

#### Standardisation and individualisation of care

Like many other HDSs, SSCSs undergo a high level of standardisation to improve efficiency [41, 42]. Analysis of the evidence from multiple methods, including relevant literature [24, 44, 50], suggests that policymakers need to focus on both standardisation and individualisation of care where appropriate to minimise undesired outcomes such as OTDSCs. The combination of unfavourable working conditions (e.g., high workload and work pressures) and a high level of standardisation of care (protocols, checklists and standard clinic consultation time) limits PONs’ ability to deliver care for patients and consequently increases the numbers of OTDSCs related to pre-operative care (i.e., category one).

#### Influential actors and decision-makers

Analysis reveals that PONs are critical in minimising OTDSCs related to pre-operative care. However, the following three types of actors initiate OTDSCs: clinicians reviewing patients before surgery (i.e., deeming patients to be unfit for surgery), managers and administrators (i.e., because of unavailability of resources), and patients (i.e., for reasons such as DNA and withdrawal of consent). Organisational interventions aimed at minimising OTDSCs thus need to focus on creating effective feedback processes and structures to facilitate inter-professional collaboration and knowledge exchange between the actors who play critical roles (i.e., PONs) in preparing patients for surgery, and decision-makers who initiate different types of OTDSCs.

Feedback from expert practitioners suggested interventions should focus on improving close collaboration between anaesthetists and PONs, improving knowledge transfer, and minimising OTDSCs for clinical reasons. The feedback from experts and administrators highlight that follow-up calls from nurses a few days before surgery also provide an opportunity to understand patients’ communication needs before surgery and can be a useful intervention to minimise OTDSCs for patient reasons.

#### Reporting tool to support minimising OTDSCs

**Fig. 2:**
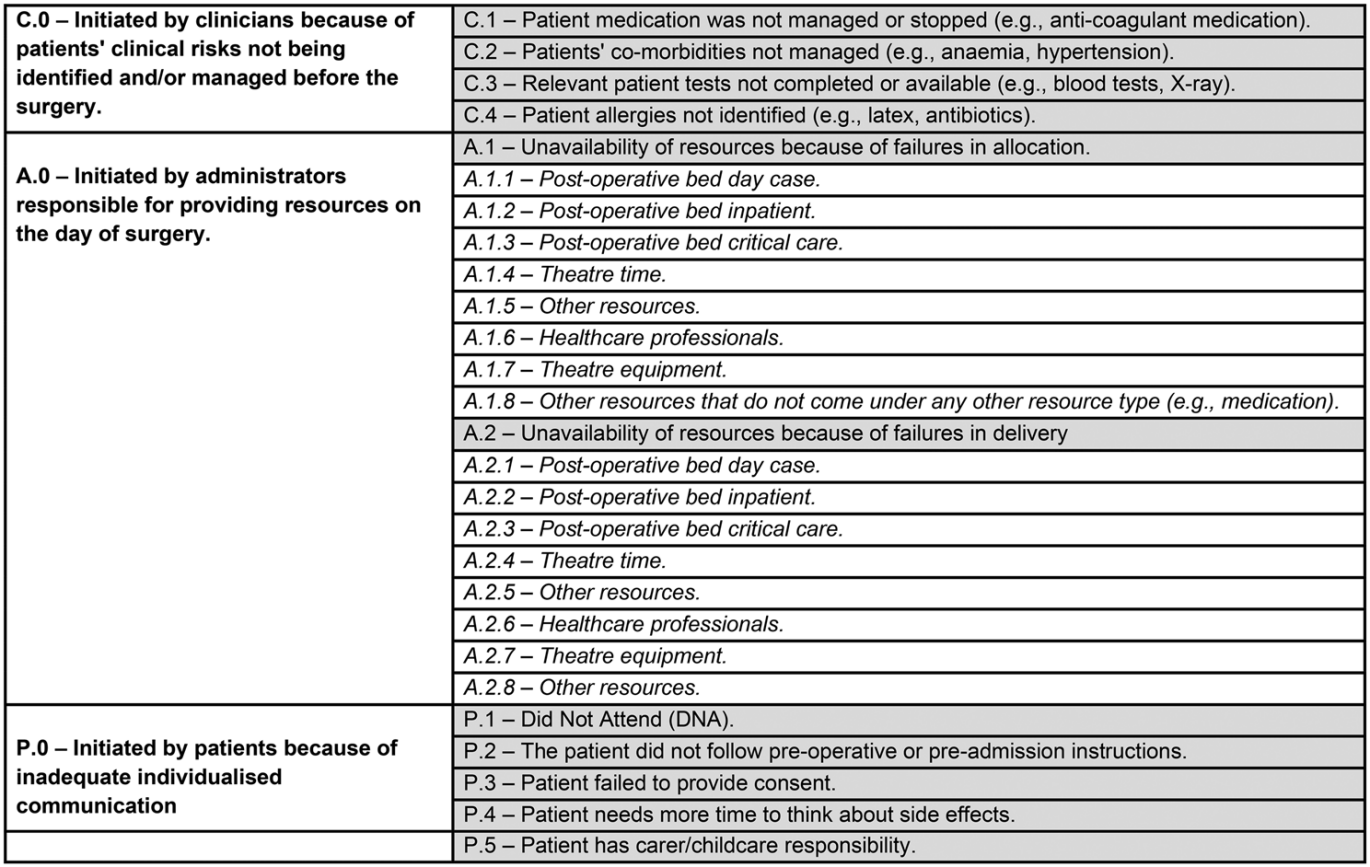
OTDSC reporting tool based on who initiated t

Despite the complexity, most of the OTDSCs are avoidable and reviewing OTDSCs in their local contexts is one of the most important to identify and design interventions to minimise OTDSCs. The findings found that the number of different reasons given for OTDSCs ranged from 15 to 76. On some occasions, reported reasons for OTDSCs varied significantly within organisations. Such variation and confusion among organisations and studies about categorising OTDSCs is a barrier to developing evidence to minimise OTDSCs and compare OTDSC rates within and outside a given organisation. Most importantly, the inability to categorise OTDSCs misses the opportunity to identify areas of improvement in the surgical care pathway. To overcome the limitations and to consider the complexity around OTDSCs, an OTDSC reporting tool was developed and is highlighted in Fig. 2.

In line with our analysis and the taxonomy of OTDSCs (Fig. 1), the categorisation consists of three actors who initiate OTDSCs: clinicians (C.0), administrators (A.0) and patients (P.0). OTDSCs initiated by clinicians were divided into four reasons (C.1 to C.4), while patient-initiated OTDSCs were divided into five reasons (P.1 to P.5). Administrator-initiated OTDSCs (i.e., unavailability of resources) can be further divided into two main groups: failure to allocate resources in preparation of patients for surgery (A.1) and delivery of resources (A.2). Each branch is further divided into eight branches or reasons depending on the resource (A.1.1 to A1.8 and A.2.1 to A.2.8).

The evidence-informed OTDSC reporting tool has many advantages, enabling categorisation of OTDSCs in a meaningful and systematic way to improve specific organisational processes, differentiating preparing patients for surgery with care delivery problems (i.e., category two) from failure to prepare patients for surgery (i.e., category one) in the following areas: care planning, care communication and scheduling. For example, OTDSCs resulting from resource unavailability because of resource delivery problems related to capacity and demand or patient flow are identified in A-2. In contrast, failure to allocate resources can be identified under A.1. Where necessary, using sub-categories, managers can identify specific issues such as failure to allocate theatre time (A.1.4) or post-operative critical care bed type (A.2.3). Where necessary, hospitals can use the tool as a framework and adapt it by adding further sub-categories, such as different types of unavailability of healthcare professionals, beds or equipment based on local contexts.

### Strengths and limitations

OTDSCs significant problem but very little is known about how to minimise them. The study developed four IPTs that can be used to minimise OTDSCs and used by other researchers. However, developing IPTs in a realist investigation requires dealing with a breadth of information related to the enquiry. Using multiple methods provided the advantage of overcoming the limitations identified in OTDSC literature and understanding the phenomenon from multiple perspectives from different actors. The ability to access direct feedback from experienced administrators, practitioners and patients was useful in gaining and validating insights and developing IPTs. We found that using the IACMO heuristic tool helped develop and present theories with better clarity, as it demonstrates the inner workings of interventions and enables actors to interact with contexts to activate mechanisms that produce outcomes. The IPTs assist in actualising the next phase of our study, in which these IPTs will be refined.

OTDSCs are considered a failure on several levels (e.g., policy, system, organisational, professional, speciality) and are politically sensitive subjects. Full anonymity was guaranteed to participants to encourage confidentiality and build trust, making it difficult to disclose their roles and where they work. The IPTs were formed in an iterative process, moving back and forth between the data. However, using multiple methods is a time-consuming and resource-intensive process, particularly when only one person completes the analysis [26]. As with all evidence reviews, the robustness of these findings is limited by the quality of the data. Since this review was conducted as part of a PhD project interrupted by the COVID-19 pandemic over a long period, some of the literature searches could be considered outdated. A further possible weakness in the realist evidence synthesis is that these findings have been derived from the researcher’s interpretation of the data, and other researchers may interpret the data differently [54]. The study authors have mixed healthcare management and health services research management backgrounds but have endeavoured to remain as objective as possible during the analysis process.

## Conclusion

OTDSCs are a longstanding global challenge that continues to create significant suffering for patients and waste in health systems but most literature on OTDSCs is descriptive and atheoretical. There is an urgent need to why OTDSCs are common and to understand how to minimise OTDSCs. The theory building exercise suggest that despite their complexity and high prevalence, OTDSCs are largely preventable.

We found four main IPTs that describe how and why different types of OTDSCs due to patients are deemed unfit for surgery, patient DNA or refusal to undergo surgery and resource unavailability. The IPTs highlighted the tension between the standardisation and individualisation care in interventions used for the care planning, communication and delivery initiated OTDSCs. The IPTs suggest that OTDSCs can be minimised by preparing patients undergoing surgery carefully, considering various aspects of individualisation of care such as clinical care planning, communication and resource allocation and delivery. The IPTs can be used to gain insights into designed various interventions to minimise OTDSCs.

Strengthening pre-operative preparation for patients undergoing surgery and providing the required support to PONs is critical for minimising most OTDSCs. The study identified that failures in various aspects of individualised care in preparing for surgery could contribute to OTDSCs, and developed a taxonomy and reporting tool which have practical implications for policymakers and practitioners. In the next phases of realist investigation, using empirical data, we will aim to understand how, why and in what contexts minimisation of the tension between standardisation and individualisation of care can take place, enabling them to work together to minimise OTDSCs.

## Data Availability

The datasets generated and/or analysed during the current study are not publicly available due to the requirement for anonymisation and confidentiality. The data extracted from the literature is available from the corresponding author at reasonable request.

## Abbreviations

CMO: Context–Mechanism–Outcome
DNA: Did Not Attend
GA: General Anaesthetic
HCP: Healthcare Professional
HDS: Healthcare Delivery System
IPT: Initial Programme Theory
MMAT: Mixed Method Appraisal Tool
OTDSC: On-The-Day Surgery Cancellation
OT: Operating Theatre
PON: Pre-Operative Nurse
SSCS: Scheduled Surgical Care System
